# The High “Cost” of Experimental Drugs Obtained Through Health Litigation in Brazil

**DOI:** 10.3389/fphar.2020.00752

**Published:** 2020-05-19

**Authors:** Ricardo Eccard da Silva, Elisangela da Costa Lima, Maria Rita C. G. Novaes, Claudia G. S. Osorio-de-Castro

**Affiliations:** ^1^Office of Clinical Trials, Brazilian Health Regulatory Agency (Anvisa), Brasília, Brazil; ^2^School of Pharmacy, Federal University of Rio de Janeiro, Rio de Janeiro, Brazil; ^3^Faculty of Health Sciences, University of Brasília, Brasília, Brazil; ^4^School of Medicine, Health Sciences Education and Research Foundation, Brasília, Brazil; ^5^Sergio Arouca National School of Public Health, Oswaldo Cruz Foundation, Rio de Janeiro, Brazil

**Keywords:** litigation, clinical trials, compassionate use, expanded access, adverse drug event

## Abstract

**Background:**

Brazilian patients have legal right to access unlicensed medicines undergoing clinical research, if there is evidence of efficacy and safety. This study investigated the occurrence of serious adverse events related to very high-cost medicines from clinical studies, expanded access and compassionate use programs, obtained by patients though health litigation.

**Methods:**

A descriptive study using secondary data investigated unlicensed medicines obtained through lawsuits from 2010 to 2017, costing more than 1 million Brazilian reais (BRL), adjusted by the Brazilian Consumer Index to July 2017. Data sources were the Brazilian Health Surveillance Agency Registry (DATAVISA) and Adverse Events in Clinical Studies (NotivisaEC) Databases. Medicines were categorized by the Anatomical Therapeutic Chemical classification to level 03 and events by the WHO Adverse Drug Reaction Terminology. The study received ethical approval by the University of Brasilia Institutional Research Board.

**Results:**

In the period, 812 drugs were obtained through litigation, and of these, 78 exceeded cost of 1 million BRL; 44 of them presented reports of 1,248 serious adverse events. Total Brazilian Government expenditure with these drugs was 3.2 billion BRL. Class L04A (n=7) showed greater expenditures (over 1.8 billion BRL). One hundred ninety-six deaths occurred and L01X was the most involved category (49.5%). Most other serious events (n=419) and sequelae (n=10) were related to L01X.

**Conclusion:**

Very high-cost drugs paid for by the government and obtained through health litigation presented deaths and serious adverse events in expanded access and compassionate use programs in Brazil.

## Introduction

Serious and/or rare conditions with high social impact and risk of death are usually treated with high-cost drugs either recently brought to market or still in trial for later licensing (OPAS, 2009). However, these products may worsen a patient's condition if their efficacy and safety are not known or acceptable.

In Brazil, as in other countries, patients with serious clinical conditions with no therapeutic alternative may have access to unlicensed products that are still under development through compassionate use and expanded access programs (Mosegui and Antoñanzas, 2019). Authorizations for the expanded access program are given to groups of patients and the drugs must necessarily be in a phase III clinical trial (Brasil, 2013a). On the other hand, entry into the compassionate use program, which is individualized, may be authorized when the medicine is at any phase of clinical development (Brasil, 2013a). Since 2012, sponsors of clinical trials are further obligated to provide participants with the medicine under investigation free of charge, even after the study's conclusion, for as long as there is a clinical benefit (Brasil, 2012).

Patients included in clinical trials and in compassionate use and expanded access programs must be monitored (Brasil, 2013a; Brasil, 2015) for the occurrence of serious adverse events (Brasil, 2015). Unexpected events under suspicion of cause by the experimental drug must be notified to the *Agência Nacional de Vigilância Sanitária* (Brazilian Health Regulatory Agency, Anvisa). Data from these notifications are important when evaluating the health profile of the medicines under investigation, especially those used for rare conditions, for which there is relatively little evidence available.

However, patients also access unlicensed medicines—whether or not they are included into the programs discussed above—through litigation. Lawsuits have been filed in Brazilian courts to force the provision of experimental treatments, based on medical prescriptions issued to the plaintiffs (Diniz et al., 2014). In Brazil, judicialization has encompassed not only medicines, but also health products and other technologies (Diniz et al., 2014), with increasingly higher expenses (Ministério da Saúde do Brasil, 2018a; Ministério da Saúde do Brasil, 2018b; Ministério da Saúde do Brasil, 2018c).

Despite the dissemination of Brazilian studies on the provision of medicines through court rulings, there is scarce literature on the risks and clinical outcomes of this type of provision (Teodoro et al., 2017). This study analyzed serious adverse events notified during clinical trials, compassionate use, expanded access, and post-trial provision of experimental drugs that were the object of lawsuits filed against the Ministry of Health (MoH), with an adjusted cost of over one million reais, in the period from 2010 to 2017.

## Methods

We carried out a descriptive study based on independent secondary databases.

We obtained, from the *Sistema Eletrônico do Serviço de Informação ao Cidadão* (Citizen Information Service Electronic System, e-SIC) (Brasil, 2019), a list of materials (medicines, health products, foods and others) acquired by the MoH, in the period from January 2010 to July 2017.

Following this step, we proceeded to identify which purchases involved drugs demanded through litigation but which had also been used in clinical trials (including post-trial provision), expanded access or compassionate use programs, made at any point during the study period with a contract value equal to or greater than 1 million BRL. The medicine prices listed in the purchase were adjusted for July 2017 by the *Índice Nacional de Preços ao Consumidor Amplo*—IPCA (National Broad Consumer Price Index) (IPCA, 2019). Expenses were calculated for all purchases of the same medicine and added.

The resulting list of costly medicines obtained through litigation was then screened for their safety profile during use in clinical trials, expanded and compassionate use programs in Brazil through both the *Sistema de Controle de Pesquisa Clínica* (Clinical Trial Control System, SCPC) and the *Sistema de Notificação de Eventos Adversos Graves em Ensaios Clínicos* (Adverse Events in Clinical Trials Notification System, NotivisaEC) to verify existence of adverse events reports (Anvisa, 2018). Drugs for which there were no reports of serious adverse events were excluded.

Drugs were then screened by the *Sistema de Produtos e Serviços sob Vigilância Sanitária* (Products and Services under Health Surveillance System, Datavisa) to verify the existence of a valid license.

We consulted the *Denominação Comum Brasileira* (Brazilian Non-proprietary Drug Name, DCB) and the commercial names of all drugs that had been licensed as medicines. For drugs not included in the DCB, synonyms were obtained from a commercial platform (Cortellis, Clarivate Analytics) (Clarivate Analytics, 2018), which works with updated data regarding pharmaceutical products, clinical trials and patents, and is used by Anvisa. For each drug we also verified its recommended clinical use based on the current International Classification of Diseases (ICD-10) (WHO, 2016).

Drugs were then classified according to the Anatomical Therapeutic Chemical classification (WHO, 2019). In order to maintain confidentiality of the drug and of commercial names of medicines, as is required by Anvisa, we chose to aggregate them at the ATC 3^rd^ level category.

Quantitative adverse events data from clinical trials, expanded and compassionate use programs were retrieved from SCPC and from NotivisaEC. Adverse events with the same WHO Adverse Drug Reaction Terminology (WHOART) (WHO, 2018a), same beginning and end dates and on the same patient were considered only once. We compiled the recorded causality data between the adverse event and the suspected drug or medicine, which was evaluated by the clinical trial's investigator or the program physician, in accordance with the International Conference on Harmonisation—ICH (ICH, 2003) and the World Health Organization (WHO, 2018b).

Microsoft Excel (version 2016) was employed to produce a database with the following information: drug name, DCB, synonyms, commercial name (if applicable), medicine license, use in clinical trial (and phase)/programs, recommended clinical use (ICD-10), adverse event notification, seriousness and type of event (expected and unexpected) and event outcome.

Results were described by sums (number of medicines, ATC 3^rd^ level, total expenditure from litigation in the period, deaths, sequelae, and number of serious adverse events).

This study was approved by the Ethics Review Board of the School of Health Sciences of the University of Brasília (CAAE: 55035116.6.0000.0030).

## Results

From January 2010 to July 2017, the MoH acquired 812 medicines in compliance with court orders, of which 78 were responsible for purchases that exceeded 1 million BRL. At July 2017 prices, these expenses totaled 5,522,033,488.00 BRL. Of these, 20 were not included in clinical trials or compassionate use and expanded access programs and 14 were not associated with adverse events. The final sample included 44 medicines (56.4%) distributed according to ATC 3^rd^ level (**Table 1**), which together cost, approximately, 3.2 billion BRL in the period (or around 58% of the expenses).

**Table 1:** Number of experimental drugs demanded through litigation, ATC 3^rd^ level classification, incurred expenditures (BRL) and related outcomes.

n	ATC 3rd level (class name)	Total expenditures with litigation^*^ (BRL)	Outcomes from clinical trials, expanded access and compassionate use programs^#^		
			Deaths	Sequelae	Other serious adverse events
7	L04A (Immunosuppressants)	1,829,315,680.96	12	7	108
3	A16A (Other Alimentary Tract and Metabolism Products)	1,174,512,692.82	16	1	58
20	L01X (Other Antineoplastic Agents)	134,340,122.74	97	10	419
1	C10A (Lipid Modifying Agents, Plain)	21,471,959.74	0	0	2
1	L02B (Hormone Antagonists and related Agents)	14,074,500.68	21	6	85
1	B06A (Other Hematological Agents)	6,804,103.46	0	0	1
1	R03D (Other Systemic Drugs for Obstructive Airway Diseases)	4,098,056.66	0	0	5
2	L01B (Antimetabolites)	3,418,587.19	12	0	20
2	J05A (Direct Acting Antivirals)	3,132,467.77	3	0	8
1	L01X (Other Antineoplastic Agents) and L04A (Immunosuppressants)	2,531,431.68	21	16	235
1	H05A (Parathyroid Hormones and Analogues)	2,460,970.36	3	1	5
1	S01L (Ocular Vascular Disorder Agents)	2,355,940.47	2	0	8
1	B02B (Vitamin K and other hemostatics)	2,169,034.39	4	1	11
1	A10A (Insulins and Analogues)	1,782,023.30	5	0	44
1	H01A (Anterior Pituitary Lobe Hormones and Analogues)	1,100,605.97	0	0	1
**44**		**3,203,568,178.21**	**196**	**42**	**1010**

Brazilian Ministry of Health and Anvisa, 2010–2017.

Source: *Litigation expenditures provided by the Brazilian Ministry of Health; ^#^Serious adverse events reported data provided by Anvisa.

In the study period, class L04A was associated with the highest expenses, at more than 1.8 billion BRL, followed by classed A16A (1.7 billion) and L01X (134 million).

The 44 drugs caused 1,248 serious adverse events. Of these, 861 (69%) took place in clinical trials, 250 (20%) in expanded access programs, 100 (8%) in compassionate use programs, and 37 (6%) were associated with post-trial drug provision (non-tabulated data).

The most numerous class was L01X (other antineoplastic agents), with 20 different drugs. This class was further represented by an association with an immunosuppressant drug (class L04A). Class L04A had seven drugs (in addition to the one in combination with L01X).

The highest number of total reported outcomes (n=526; 42.2%) contained drugs classified as L01X. L01X + L04A was second in number (n = 272; 21.8%); the medicine formed by the association had 235 (23.3%) notified serious events. L04A alone accounted for 127 (10.2%) of outcomes. Events with sequelae (n=42) were more present among users of the associated L01X/L04A medicine (16), followed by users of L01X (10) and L04A (7).

With regard to type of event, 661 (53%) were considered unexpected (data not presented in **Table 1**). The 44 medicines were suspected of causing 196 deaths (16% of the total of adverse events). The class most involved with this event was L01X (n=97; 49.5%), followed by two unique medicines from class L02B and by the association L01X and L04A. Each was suspected of causing 21 deaths. Class A16A, the second in expenses and represented by three medicines, was associated with 16 deaths (8.5%).

## Discussion

This study showed that 44 medicines involved in trials and expanded access or compassionate use programs and bought by the Federal Government in compliance with court orders represented 3.2 billion BRL in expenses with individual purchases between January 2010 and July 2017. These medicines had serious adverse event notifications, including 196 deaths. Unexpected events corresponded to more than 50% of observed events.

There are no accessible and transparent databases that characterize the benefit of expanded access and compassionate use of experimental drugs in South American countries (Mosegui and Antoñanzas, 2019). In Brazil, clinical trials and expanded access and compassionate use programs are regulated by Anvisa to guarantee enrolled patients' safety. They undergo prior assessment and authorization depends on the accrual of medical reports and drug safety and efficacy information which supports its prescription (Teodoro and Caetano, 2016). One of the criteria supposedly taken into consideration is the risk/benefit analysis of the demanded medicine. To participate, patients or their legal representatives must sign an informed consent document which must present the justification for product use, possible discomfort and risks, including adverse events, and expected benefits (Brasil, 2013a; Darrow et al., 2015; Teodoro and Caetano, 2016). These patients are monitored for adverse events.

However, despite the precautions taken by the regulatory agency, expanded access presumes the use of a drug of which little is known, and additionally, outside the investigative domain, which generates great uncertainty (Goldim, 2008). In these programs and trials, the availability of information and the evidence regarding the drug´s safety and efficacy are limited. Phase I and II clinical trials, for example, may not show clinical benefits, with little knowledge available for decision-making. Often, the only assessment that is possible is whether or not the experimental drug poses a greater risk than the disease itself. When access results from litigation, the situation is even more complex. It is possible that the structure for patient support and monitoring present in clinical trials may not be available. Additionally, it is not possible to guarantee that the available information will be offered to patients, or that they or their legal representatives will have a clear understanding of potential risks and harms based on a consent form.

Patients who demand to participate in expanded access and compassionate use programs are especially vulnerable because they have serious diseases, with no therapeutic alternatives, and their capacity to perceive treatment risks is compromised. These patients are far more compelled to obtain access to an experimental treatment than to take into consideration the unpredictable risks of an unlicensed drug (Darrow et al., 2015).

Despite this situation, the occurrence of serious events seems only rarely to affect the development of expanded access programs. An analysis of 11,000 requests received by the FDA to include patients into expanded access programs over the course of 10 years showed that only two programs were temporarily interrupted due to the occurrence of adverse events (Jarow et al., 2016).

There is considerable pressure on health professionals and regulatory agencies to incorporate new products, a situation which is not exclusive to Brazil. The speed with which evidence is produced and the pressure for incorporation discourage a conservative approach to the use of technologies. Rapid- and slow-acting insulins, for example, which are leading objects of lawsuits against SUS, were incorporated, even after several studies that showed the lack of gains in effectiveness from the system's perspective (De Souza et al., 2014; Laranjeira et al., 2016; Chieffi et al., 2017; CONITEC, 2019a; CONITEC, 2019b).

In the face of health litigation, the health system has at times been called upon to provide technologies with no scientific evidence in support of the proposed use, or with fragile evidence of benefits amid a context of growing costs (Figueiredo et al., 2010). According to the MoH, between 2010 and 2016, the costs of litigation for the federal government increased more than 10-fold. In 2016, the expenses with judicial health demands consumed 1.3 billion BRL of the federal budget. The list with the ten most expensive medicines was responsible for 90% of that sum (Conselho Nacional de Justiça, 2019). The expenses resulting from litigation have an impact on the medicines policy budget, with a reduction in the offer of other listed medicines, exams, and consultations to the general population (Paim et al., 2017). On the other hand, there seem to be inconsistencies and information failures even in the application of the SUS *Protocolos Clínicos e Diretrizes Terapêuticas* (Clinical Protocols and Therapeutic Guidelines, PCDT) (Figueiredo et al., 2013; Dias and da Silva, 2016) when regarding litigated medicines. In 2017, an audit of 414 judicial demands for medicines in Brazil showed that around half did not even include a diagnosis of the disease to be treated (Ministério da Saúde do Brasil, 2018d).

Many medicines demanded through litigation are generally prescribed for the treatment of chronic diseases, such as cancer and rare diseases (Chagas and Santos, 2018). In this study, a large portion of the medicines was classified as antineoplastic agents (L01X, for example). In fact, this subjacent risk profile, related either to the seriousness or to lack of knowledge as to the underlying disease also draws attention to the safety problems surrounding treatment.

In 2019, the Brazilian Supreme Court decided that States cannot be compelled to provide experimental drugs. However, in exceptional cases, it is possible for courts to award access to unlicensed drugs if there is an unreasonable delay in Anvisa's appreciation of the licensing documentation (Brasil, 2016) and when three criteria are met: i) existence of a licensing request (excepting orphan drugs for rare and ultra-rare diseases); ii) existence of a license for the medicine from a renowned foreign regulatory agency; and iii) non-existence of a licensed therapeutic substitute in Brazil (STF, 2019). In any case, it is worth noting that licensing requirements in Brazil became more flexible in 2013 (Decree 8.077) (Brasil, 2013b). Furthermore, there is pressure in favor of accepting licenses from foreign agencies, which are also under pressure from the pharmaceutical industry (Osorio-de-Castro et al., 2015), weakening safety requirements for licensing.

Licensing, as a country's first medicines selection process, would be considered an initial filter to the entry of safe and effective new technologies. However, it is worth noting that lack of safety information is also a problem for medicines that have undergone the licensing stage. This has to do with the quality of the process. In France, a 2017 analysis showed that 45 out of 92 new licensed medicines were products with no therapeutic advantage and 15 new medicines were classified as unacceptable (Prescrire International, 2018). In Brazil, an analysis that employed the same criteria concluded that 82% of new medicines approved for licensing by Anvisa between 2004 and 2016 were not innovative and had low therapeutic value (Hoefler et al., 2019).

This study showed that of the 44 medicines that were used by litigants, 81% were associated with serious adverse events other than sequelae (3.4%) or death (15.7%). However, there is no way to establish if litigants suffered these same effects, and with the same severity. Nonetheless, of all outcomes 83.2% were associated with the following medicine classes: L01B (antimetabolites), L01X (other antineoplastic agents), L02B (hormonal antagonists and other related agents), L04A (immunosuppressants). We must consider that these events may have been influenced by the patients' serious clinical conditions, in addition to their comorbidities, since the drugs in these classes are potentially the last available therapeutic alternative. However, we should consider that beyond the medicine and the patient's intrinsic safety issues, the use of experimental drugs outside the research setting and as a result of litigation without the necessary controls considerably increases risks (Vidal et al., 2017).

If, on the one hand, these medicines have an inconclusive risk/benefit profile, on the other, they represent considerable expenses for the system. The medicines in classes L01 (antineoplastic agents), L02 (hormones), and L04 (immunosuppressants) are among the most expensive and most often incorporated since 2012. An analysis of expenses with medicines incorporation showed that class L corresponded to a greater variation in expenses between 2006 and 2013, with a 20-fold increase, while class L04 increased 250 times in the Brazilian federal government expenses (Luz et al., 2017). This medicine class was also responsible for high expenses in the United States, United Kingdom, Australia and Canada (Alves et al., 2018).

Class A16A (other alimentary tract and metabolism products) encompasses countless enzymes and biological medicines, many of which have been incorporated since 2014 (CONITEC, 2019c), with high added costs. Once a medicine is incorporated—and, therefore, a channel for government funding is established—new uses start to be suggested. Thus, we may assume that some of the recently incorporated A16A medicines are targets for prescriptions of new, unlicensed uses. The presence of this class shows the potential of experimental uses driving the costs of public provision.

A substantial, though unintentional, limitation of the study is the impossibility of defining exactly which litigated medicines were responsible for serious adverse events, including deaths. This barrier, imposed by Anvisa, interferes to some extent with regulatory activities (though Anvisa has the means to extract this information independently), but mainly compromises the transparency of information regarding safety of litigated medicines for assessment by the population, by patients, by health workers and by the law. This is new information which, to our knowledge, has not been previously published.

Otherwise we did not mean to establish a direct correlation between the use of drugs and the effect on individual patients. We worked with aggregated data and were not able to access patient-level data. Another possible limitation concerns the data collection strategy, since the databases were not linked. A study that used linked information, with patient-level data, would give greater robustness to the analysis.

But we do have a blueprint of adverse effects caused by these drugs. Litigation is actively supplying drugs without careful review and judges only adhere to the plaintiff´s clinician. This has been demonstrated over the years, as a process that does not favor evidence-based use of medicines in clinical practice (Lopes et al., 2010; Chieffi et al., 2017).

It is important to consider that these are mostly drugs used in severe clinical conditions with a low benefit-risk ratio but in many situations, they are needed by the patient. Clinicians acknowledge the potential safety problems in view of the dire need for treatment in many cases. Despite this, we highlight these are dangerous drugs and follow-up, through litigation is, at present, non-existent. This constitutes a paradox, since monitoring is mandatory in clinical trials, expanded and compassionate use programs in Brazil (Brasil, 2013a; Brasil, 2015).

## Conclusion

The study showed the existence of serious adverse events, including deaths, related to litigated medicines in the country, from 2010 to 2017. These medicines, grouped at the 3^rd^ level ATC, represent considerable expenses for the Ministry of Health. It is estimated that, given the reach of health litigation in the country, serious events and deaths may also be happening in connection to medicines purchased by other Federal Government institutions and at other government levels.

We point to the important implications related to the lack of transparency of data regarding the safety—or lack thereof—of experimental, unlicensed drugs, which may be obtained through litigation, which, as is known, is a fast, potentially uncritical and considerably widespread practice for accessing new medicines or new medicine uses in the country. As several countries in Latin America are also going through health litigation for access to medicines, the results presented in this paper may be useful for further discussion on drug safety issues.

## Data Availability Statement

The datasets generated for this study are available on request to the corresponding author.

## Ethics Statement

The studies involving human participants were reviewed and approved by Ethics Review Board of the School of Health Sciences of the University of Brasília. Written informed consent from the participants' legal guardian/next of kin was not required to participate in this study in accordance with the national legislation and the institutional requirements.

## Author Contributions

RS, EL, MN, and CO-D-C made substantial contributions to conception, design and acquisition of data. In addition, RS, EL, and CO-D-C analyzed and interpreted the data. All authors read and approved the final manuscript.

## Conflict of Interest

The authors declare that the research was conducted in the absence of any commercial or financial relationships that could be construed as a potential conflict of interest.
